# Performance of *Trichopria drosophilae* (Hymenoptera: Diapriidae), a Generalist Parasitoid of *Drosophila suzukii* (Diptera: Drosophilidae), at Low Temperature

**DOI:** 10.1093/jisesa/ieaa039

**Published:** 2020-05-27

**Authors:** Fernanda Colombari, Lorenzo Tonina, Andrea Battisti, Nicola Mori

**Affiliations:** 1 Department of Agronomy, Food, Natural Resources, Animals and Environment, University of Padova, Legnaro (PD), Italy; 2 Department of Biotechnology, University of Verona, Verona, Italy

**Keywords:** longevity, parasitism, biocontrol, cold adaptation

## Abstract

Survival and parasitism activity of *Trichopria drosophilae* Perkins adults, a cosmopolitan parasitoid of *Drosophila* spp., were studied under laboratory conditions using five constant temperatures at the lower range known for this enemy, from 4 to 20°C in 4°C increments. *Drosophila suzukii* Matsumura, an invasive pest of small fruits, was used as a host. Commercially available adult parasitoids were provided with 1) food and *D. suzukii* pupae; 2) food and no *D. suzukii* pupae; 3) no food and no pupae. The results show that adult females of *T. drosophilae* lived longer than males, and both generally benefitted from food supply. The highest level of survival was observed between 8 and 12°C for fed insects, irrespective of whether they were offered host pupae or not. The absence of food led to the highest mortality, but the parasitoid demonstrated considerably resistance to prolonged starvation. Successful parasitism increased steadily with temperature and reached the highest value at 20°C. Conversely, *D. suzukii* emergence rate was high after exposure of pupae to parasitoids at 4°C, while pupal mortality increased strongly with temperature until 12°C. The findings indicate that *T. drosophilae* is well adapted to the relatively cold conditions experienced in early spring and in autumn or at high elevations, when the host pupae could be largely available. The long lifespan of the adults and the ability to parasitize the host at low temperature make *T. drosophilae* potentially useful for the biocontrol of *D. suzukii*.

*Drosophila suzukii* Matsumura (commonly known as spotted wing drosophila, hereafter SWD) is an invasive pest of cherries and small fruits for which biological control has been attempted (Lee et al. 2019). Many studies have recently dealt with the understanding of when and where it is more vulnerable to its natural enemies (Cini et al. 2012, Wiman et al. 2016). In particular, Rendon et al. (2018) emphasized the potential of SWD to successfully reproduce at suboptimal temperatures (5–18°C), often experienced in spring/autumn or at high elevations (Tonina et al. 2016). Consequently, it is crucial to determine how parasitoid activity might be affected by the same temperature range (Hance et al. 2007).

The pupal parasitoid *Trichopria drosophilae* Perkins, a native species in the invasion range of SWD, has been recently used in biocontrol programs (Woltering et al. 2019), and Kruitwagen et al. (2018) highlighted the need to test the efficiency of the parasitoid below 20°C. The few experiments conducted so far showed that successful parasitism is possible at 12.6 and 15°C, whereas at 5°C adults survived but did not parasitize hosts (Rossi-Stacconi et al. 2017, Amiresmaeili et al. 2018, Wang et al. 2018).

To improve the knowledge on the biology of *T. drosophilae*, we investigate the longevity and survival of both sexes, and their performance, under different conditions at constant temperatures ranging from 4 to 20°C. Data obtained might clarify the potential of the biological control agent at colder sites or in cold seasons.

## Materials and Methods

Adults of *T. drosophilae* used in the experiment were commercially available insects reared on *Drosophila melanogaster* Meigen (Diptera: Drosophilidae) (Bioplanet s.r.l., Cesena, Italy) maintained at 15°C (±1°C) until the beginning of the experiments, whereas pupae of SWD were obtained from laboratory colonies reared according to Tonina et al. (2016).

Longevity of *T. drosophilae* adults and parasitism of SWD pupae were studied in a test tube (2.5 cm diameter, 8 cm length) made of transparent plastic and placed in five controlled cabinets set under long-day photoperiod (16 h light) at five constant temperatures: 4, 8, 12, 16, and 20°C (±1°C). Relative humidity (70–80%) was maintained in the test tubes using soaked cotton wool that provided also a constant source of water (Fisher and Andrés 1999) for the parasitoids of all treatments.

Adult parasitoids were progressively taken from the commercial boxes as soon as they emerged from *D. melanogaster* pupae and were randomly inserted into each test tubes (average 58, SD = 14, *n* = 30). Consequently, for mating purposes the sex ratio was male-biased (male:female average 2.68, SD = 0.95, *n* = 30) and slightly different in each tube, as we needed to test individuals of the same age (1–2 d-old female adults) with a high probability of being mated during the period of maximum egg load (Wang et al. 2016).

Groups of adult parasitoids were assigned to three treatments: 1) with food; 2) with food and host; 3) without food and host. For food, ten droplets of honey diluted with water (80% honey) were provided on a plastic strip (0.8 × 3.0 cm) and inserted into each tube (Fisher and Andrés 1999). For host, 10 pupae of SWD (up to 4 d old; Wang et al. 2016) were provided for parasitoids on a filter paper strip (2.0 × 4.0 cm) sprayed with sterile water (Zhu et al. 2017). Two replications of each treatment were carried out for a total of 30 test tubes (3 treatments × 5 temperature conditions × 2 replications).

Test tubes were checked and pupae replaced twice a week for adult parasitoid survival and performance in the first 3 mo, and then weekly until the end of the experiment, i.e., when no live *T. drosophilae* adults initially put inside the test tubes were still present (total number of checks = 26). Dead parasitoids were not replaced during the experiment. At each check, the cotton wool ball was sprayed with sterile water, and the plastic strip with honey droplets and the pupae were replaced. SWD pupae were transferred in new tubes kept at 20°C (±2°C) under the same photoperiod used in the experiment until SWD or parasitoid emergence. All individuals were then identified, counted, and sexed, and the pupal cases examined. If no exit holes had been observed, SWD pupal cases were carefully dissected and the content analyzed and classified as: 1) not fully developed or emerged *T. drosophilae*; 2) not fully developed/emerged SWD; 3) pupae dead for unknown reasons (i.e., pupae parasitized or not, as the parasitoid or the fly died in the early stages of development; Wang et al. 2016).

The variables considered in the study were: 1) lifespan of *T. drosophilae*; 2) mortality of SWD pupae caused by *T. drosophilae*; 3) unparasitized healthy pupae of SWD; 4) dead pupae of SWD for unknown reasons. The lifespan of *T. drosophilae* was obtained from periodical check of the number of live *T. drosophilae*. Pupal mortality of SWD caused by *T. drosophilae* was based on the sum of *T. drosophilae* adults emerged (or SWD pupal cases with a parasitoid emergence hole when adult parasitoids were not easily countable) and pupal cases containing undeveloped *T. drosophilae*. Unparasitized healthy pupae of SWD was based on the sum of SWD adults emerged or pupal cases with a SWD emergence hole and of pupal cases containing undeveloped SWD. Lastly, pupae dead for unknown reasons were obtained from the pupal cases without emergence holes and pupal cases with a content not identifiable.

As our experimental design considered weekly cumulative mortality, nonlinear regressions with a 95% confidence interval including the two replicates of each trial were used. A three-parameter sigmoidal equation [*y* = *a*/(1 + e−(*x* − *x*0)/*b*)] was run to calculate the 50% of survival and to plot the temperature-dependent survival curves. Regressions and related statistical analyses were performed and plotted with the software Sigmastat 3.1 and SigmaPlot 11.0, respectively (SSPS, Inc., Point Richmond, CA). Linear models was used: 1) to test the differences in the temperature-dependent survival curves, with temperature, treatment and sex as fixed factors, and all possible interactions among factors; 2) to examine the effects of temperature (categorical fixed factor), *T. drosophilae* survival (continuous fixed factor) and their possible interactions on the outcome of the pupal development. The models were performed using the packages ‘nlme’ for general mixed-effects models (Pinheiro et al. 2018) implemented in R (version 3.0.2—R Core Team 2014). The assumptions of the models were evaluated by visual inspection of the diagnostic plots of the model residuals.

## Results

**Fig. 1.**
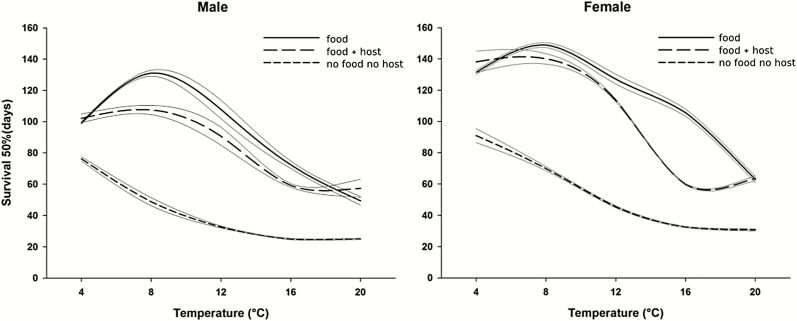
Temperature-dependent survival curves of male (left) and female (right) *Trichopria drosophilae* from three treatments (food, i.e., honey; food and host, i.e., honey and pupae of SWD; no food and no host, i.e., no honey and no pupae of SWD). Each curve was plotted using the 50% survival time. Gray lines represent the 95% confidence interval for each regression curve.

Both male and female *T. drosophilae* generally lived longer when supplied with food than when offered food and host pupae or when they were deprived of both food and host (Table 1, Fig. 1). The highest level of survival was observed between 8 and 12°C for fed parasitoids irrespective of the sex and host presence. Females lived longer than males, especially at low temperature (from 4 to 12°C). The low and high temperatures used in the experiment (4 and 20°C) produced similar results for the parasitoids provided with food and with food and host. Insects deprived of food and host survived longer at 4°C and then survival decreased to a stable level between 12 and 16°C.

**Table 1.** Results of the linear model used to test the effects of temperature, sex, treatment, and all possible interactions among these factors on *Trichopria drosophilae* survival

Factor and interaction	df	Sum sq.	Mean sq.	*F* value	Pr(>*F*)	
Temperature	4	35,274	8,818.4	525.3551	<2.2e^−16^	***
Sex	1	5,089	5,089.1	303.1798	<2.2e^−16^	***
Treatment	2	35,374	17,687.1	1053.7037	<2.2e^−16^	***
Temperature × Sex	4	704	175.9	10.4795	1.96e^−05^	***
Temperature × Treatment	8	5,979	747.4	44.5272	1.47e^−14^	***
Sex × Treatment	2	307	153.7	9.1582	0.000786	***
Temperature × Sex × Treatment	8	747	93.4	5.5619	0.0002349	***
Residuals	30	504	16.8			

***P < 0.001.

**Fig. 2.**
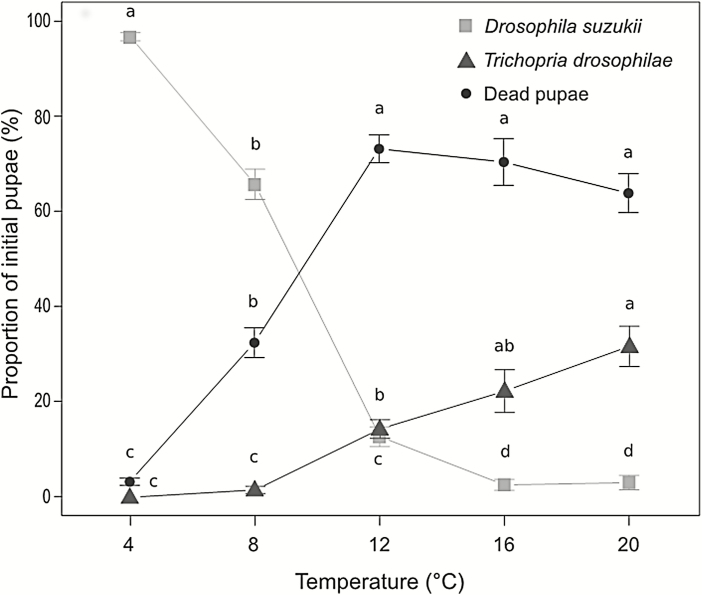
Proportion of pupae yielding SWD (squares), *Trichopria drosophilae* (triangles) and dead pupae (circles) obtained after pooling the two ‘food and host’ replications for each temperature tested. Each symbol represents the mean value ± SE (*n* = 15). Letters above the symbols indicate statistically significant differences in pairwise comparison within each variable (Tukey’s test performed following one-way ANOVA analysis, *P* < 0.05).

The outcome of pupal development (emergence of *T. drosophilae*, emergence of SWD, dead pupae of SWD) was significantly affected only by temperature, regardless of the parental age of the parasitoid (Table 2). Successful parasitism started at 8°C and then increased steadily with temperature. Conversely, SWD emergence rate was higher after exposure at 4°C and then decreased rapidly, reaching almost 0 values at temperatures between 16 and 20°C. Pupal mortality increased strongly with temperature until 12°C and then remained almost constant (Fig. 2).

**Table 2.** Results of the linear model employed to test the effects of temperature and parental age of *Trichopria drosophilae*, and their interaction, on the pupal development of SWD

Factor and interaction	df	Sum sq.	Mean sq.	*F* value	Pr(> F)	
Outcome: % of pupae producing *T. drosophilae* adults						
Temperature	4	1.04147	0.260367	28.0579	6.75e^−13^	***
Parental age	1	0.00001	0.000007	0.0008	0.9781	
Temperature × Parental age	4	0.02419	0.006047	0.6516	0.6281	
Residuals	57	0.52894	0.00928			
Outcome: % of pupae producing SWD adults						
Temperature	4	9.9966	2.49916	421.0564	<2e^−16^	***
Parental age	1	0.0032	0.00321	0.5415	0.4648	
Temperature × Parental age	4	0.0313	0.00782	1.3179	0.2743	
Residuals	57	0.3383	0.00594			
Outcome: % of SWD dead pupae (content not identifiable)						
Temperature	4	5.0397	1.25993	92.4229	<2e^−16^	***
Parental age	1	0.0157	0.01566	1.1485	0.2884	
Temperature × Parental age	4	0.0648	0.01621	1.1889	0.3255	
Residuals	57	0.777	0.01363			

***P < 0.001.

## Discussion

Adults of both sexes of *T. drosophilae* coped well with low temperature if constantly supplied with honey and water, surviving more than 4 mo and thus almost doubling the value found by Amiresmaeili et al. (2018) with sugar-fed parasitoids. Furthermore, depending on factors such as increasing temperature and/or energy-consuming activities (i.e., mating and reproduction), life expectancy declined for both males and females, although at a different rate since males lived shorter than females. As in females, a trade-off between reproduction and survival is expected in males, but little is known about balancing the costs for sperm production and finding females (Hoogendoorn et al. 2002). In our experiment, there were no costs involved in finding females: thus, we can only hypothesize the occurrence of different metabolic rates and different temperature-induced shift in resource allocation in the two sexes. This was also demonstrated by the fact that at the lowest temperature tested (4°C), when no successful parasitism occurred, there were no differences in the lifespan recorded within each sex between the treatments ‘food’ and ‘food and host’. The same result emerged at the highest tested temperature (20°C) suggesting that, if food is available, *T. drosophilae* is able to easily replace the resources allocated to reproduction before approaching the thermal optimum for its performance (i.e., 20–25°C; Rossi-Stacconi et al. 2017). Above 25°C, metabolic costs likely become too high, especially if the host is abundant, as female parasitoids can survive only 27 d at 23°C (Wang et al. 2016). As expected, the absence of food led to the highest mortality, but adults were able to cope with food deprivation as they were able to survive up to 3 mo at 4°C and up to 30 d at 20°C. As far as we know, there are no similar data on *T. drosophilae* or other parasitoids associated to *Drosophila* spp. to be used for comparison. Only Da Silva et al. (2019) assessed that adults of *Pachycrepoideus vindemmiae* (Rondani) (Hymenoptera: Pteromalidae), kept without food but with a constant supply of water, lived for about 15 d at 24°C. Nonetheless, the ability of fasting *T. drosophilae* adults to survive at low temperature might be an important trait, allowing the biocontrol agent to cope with host absence and long cold periods.

Interestingly, we observed that *T. drosophilae* can successfully parasitize SWD pupae when temperature is above 8°C, a result that lowers the threshold of 12.6 and 15°C recently demonstrated by Wang et al. (2018) and Amiresmaeili et al. (2018), respectively. At 4°C, *T. drosophilae* was not able to exert any action against the host, as demonstrated by the high percentage of pupae (almost 100%) giving rise to SWD adults. As host-feeding has not been observed for *T. drosophilae* (Carton et al. 1986), the high rate of pupal mortality (starting at 8°C, peaking at 12°C, and then showing no significant variations at 16 and 20°C) without a corresponding/proportional number of parasitoid offspring, according to Abram et al. (2019) could be explained by nonreproductive effects (e.g., pseudoparasitism). Despite the fact that, for parasitoids and for biocontrol practitioners, nonreproductive effects could be costly from many points of view, they can sometimes be the principal cause of host mortality and thus useful in the short-term. This study also showed the absence of any variation in the parasitism rate of females during their lifespan, a result that complements the study of Chen et al. (2018). In their experiment, the authors took into consideration females of *T. drosophilae* maintained without hosts but with food from 1 to 40 d. After mating, females of different ages were offered pupae of *D. melanogaster* and demonstrated an extremely high parasitism rate at all time points. In this study, we obtained the same outcome, extended up to 100 d for females kept at 12°C.

In conclusion, compared with the other parasitoids known to attack SWD in the field, our study demonstrates that *T. drosophilae* is the only species able to be active during these low temperature periods. At constant 15°C, the pupal parasitoid *P. vindemmiae* does not show any parasitization activity as well as the larval parasitoid *Leptopilina heterotoma* (Thomson) (Hymenoptera: Figitidae) (Rossi-Stacconi et al. 2017), whereas for the most part of the congeneric *Leptopilina boulardi* (Barbotin, Carton & Kelner-Pillault) (Hymenoptera: Figitidae), alongside *Asobara tabida* (Nees) (Hymenoptera: Braconidae), enter a diapause (Carton et al. 1986). However, in *A. tabida* the mechanism is more complex, being associated with other factors such as photoperiod and host species (Fleury et al. 2009). As successful parasitism has been observed at low temperature under seminatural conditions (unpublished), availability of field data is needed to support and confirm the importance of the seasonal role of the parasitoid in the population suppression of SWD.
